# Unerwarteter Tod eines zweijährigen Kindes: Wie die Elektronenmikroskopie half, der Ursache auf die Spur zu kommen

**DOI:** 10.1007/s00292-025-01535-6

**Published:** 2026-01-15

**Authors:** Thomas Menter, Dorothea Wand, Maya Caroline André

**Affiliations:** 1 Pathologie, Institut für medizinische Genetik und Pathologie, Universitätsspital Basel, Schönbeinstrasse 40, 4031 Basel, Schweiz; 2 Medizinische Genetik, Institut für medizinische Genetik und Pathologie, Universitätsspital Basel, Basel, Schweiz; 3 Abteilung für Pädiatrische Intensivmedizin und Pneumologie, Universitäts-Kinderspital beider Basel, Basel, Schweiz

**Keywords:** Mitochondriopathie, GCDH, Parakristalline Inklusionen, Autopsie, Genetische Analysen, Mitochondriopathy, GCDH, Paracrystalline inclusions, Autopsy, Genetic analysis

## Abstract

Ein 2‑jähriges Kind wurde nach kurzzeitigen gastrointestinalen Symptomen leblos im Bett aufgefunden.

Bei histologisch unauffälligen Befunden zeigten sich in der elektronenmikroskopischen Untersuchung deutliche Atypien der Mitochondrien mit linearen parakristallinen Inklusionen.

In der genetischen Abklärung zeigten sich Varianten unklarer Signifikanz in den Genen *TYMP* und *AIFM1* und eine heterozygote pathogene Mutation im Gen *GCDH*.

Bislang lässt sich keine klare Kausalität zwischen den gefundenen Mutationen und der Mitochondrienmorphologie darstellen, in In-vitro-Versuchen zeigen sich jedoch gewisse Analogien zu den hier nachgewiesenen strukturellen Veränderungen der Mitochondrien.

## Abstract (en)

A two-year-old child was found lifeless in bed after experiencing brief gastrointestinal symptoms.

Histological examination revealed no abnormalities, but electron microscopy showed clear atypia of the mitochondria with linear paracrystalline inclusions.

Genetic testing revealed variants of unclear significance in the *TYMP* and *AIFM1* genes and a heterozygous pathogenic mutation in the *GCDH* gene.

To date, no clear causality between the mutations found and the mitochondrial morphology can be established, but in vitro experiments show certain analogies to the structural changes in the mitochondria detected here.

Der unerwartete Tod eines Kindes stellt für alle Beteiligten – Eltern, Familie und auch die behandelnden Ärzte – eine große Belastung dar. Die genaue Klärung der Todesursache kann in vielen Fällen helfen, das Geschehene besser verstehen und verarbeiten zu können. Die „klassische“ Autopsie kann hierzu einen wichtigen Beitrag leisten [5].

## Fallbericht

Ein knapp 2‑jähriger Junge wurde bei ausgeprägt symptomatischer Gastroenteritis mit Erbrechen und Durchfall auf der Notfallaufnahme vorgestellt. Nach Initiierung einer symptomatischen Therapie wurde die Familie wieder nach Hause entlassen. Am nächsten Morgen fand die Mutter das Kind leblos im Bett vor. Trotz sofort eingeleiteter Maßnahmen und intensivmedizinischer Betreuung konnten bereits bei Aufnahme lichtstarre Pupillen dokumentiert werden und innerhalb weniger Stunden wurde der Tod des Kindes festgestellt. Kurz vor dem Tod wurde in der notfallmäßig durchgeführten Echokardiographie der Verdacht auf eine dilatative Kardiomyopathie gestellt. Auffälliger Laborparameter war eine deutliche metabolische Azidose ohne Laktatanstieg.

Aufgrund dieser unklaren Befunde mit Verdacht auf Kardiomyopathie oder eine Stoffwechselerkrankung wurde die Durchführung einer Autopsie mit den Eltern besprochen. Diese stimmten schließlich einer Teilautopsie des Herzens sowie einer Entnahme von Lebergewebe zu.

**Abb. 1:**
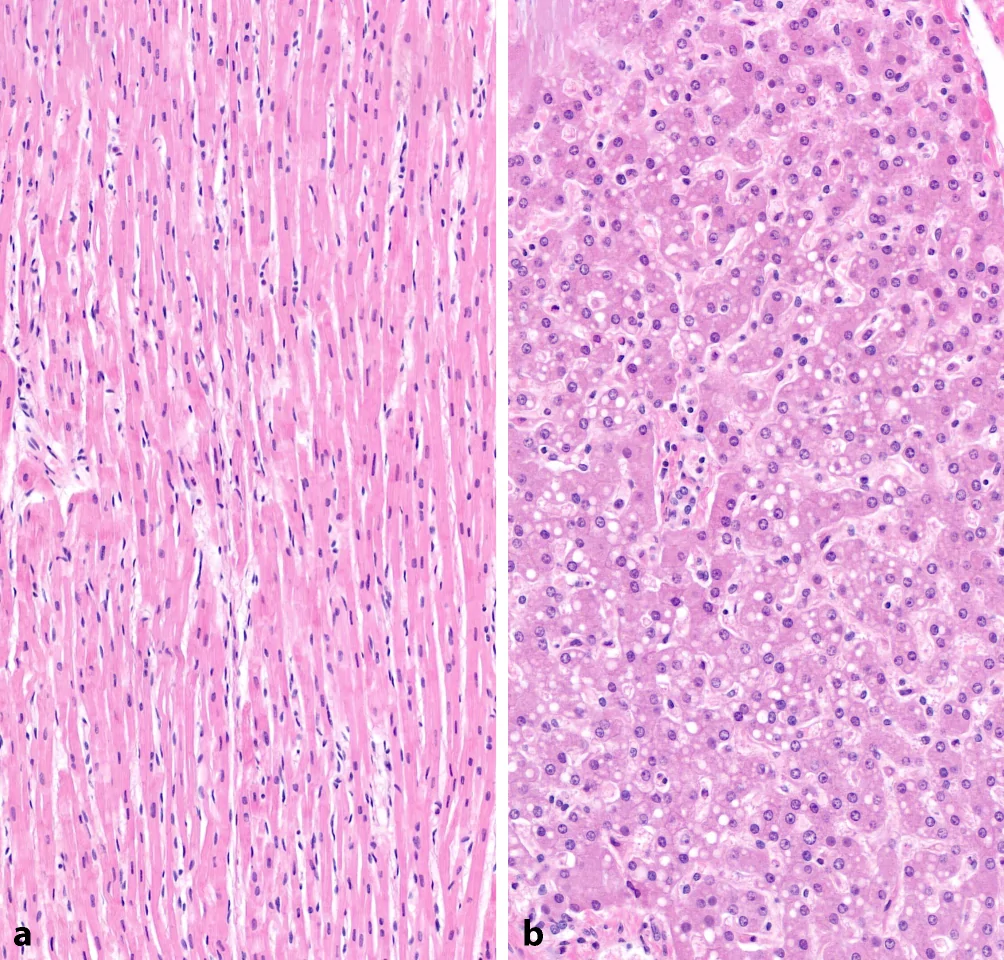
Histologie. **a** Unauffälliges Myokard ohne Nachweis einer Fibrose oder von Entzündungsinfiltraten. HE, Vergr. 100:1. **b** Leberparenchym mit diffuser mikrovesikulärer Steatose; kein Nachweis von Schocknekrosen oder interstitiellen Entzündungsinfiltraten. HE, Vergr. 100:1

Makroskopisch zeigte sich ein normal konfiguriertes Herz. Das Gewicht sowie die Maße des Herzens (Klappenumfänge, Herzwanddicken) lagen in der Norm. So konnte die echokardiographische Verdachtsdiagnose einer dilatativen Kardiomyopathie nicht bestätigt werden. Auch histologisch zeigten sich keine Auffälligkeiten des Herzens. Es fand sich eine normale Mikroarchitektur ohne Nachweis von Fibrosearealen. Auch vermehrte Entzündungsinfiltrate als Hinweise für eine Myokarditis waren nicht vorhanden (Abb. 1a).

Das Leberparenchym erschien makroskopisch etwas gelblich, histologisch zeigte sich eine mikrovesikuläre Steatose (Abb. 1b).

**Abb. 2:**
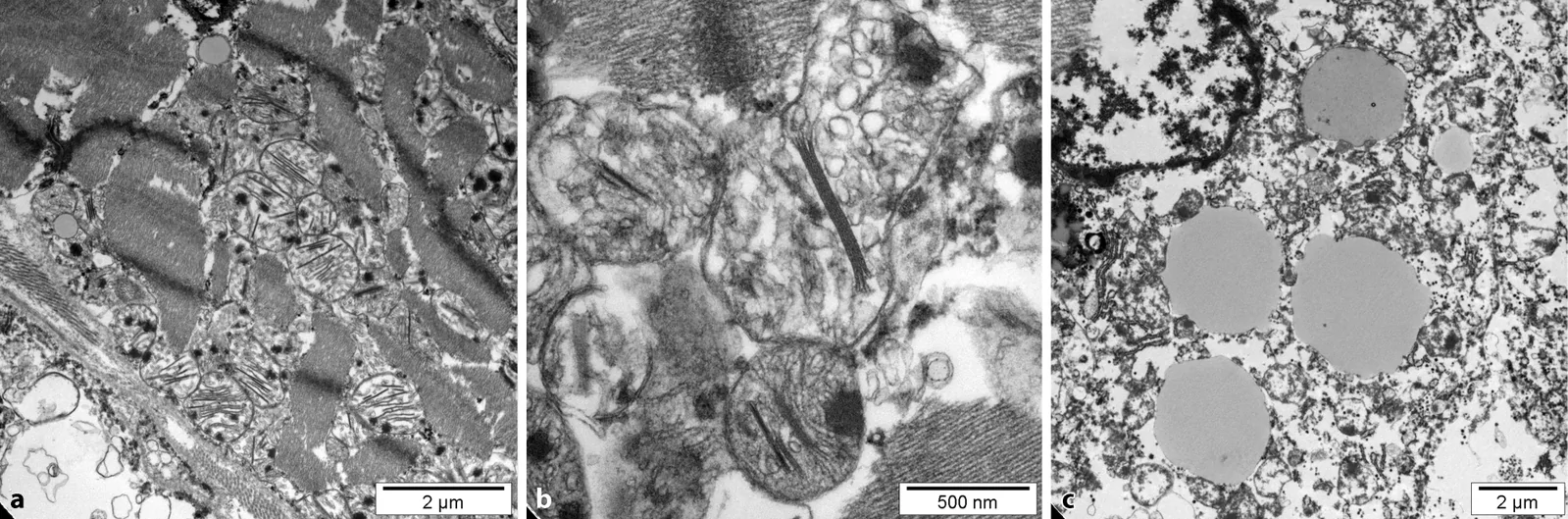
Elektronenmikroskopie. **a** Übersichtsbild des Myokards mit Nachweis deutlich vermehrter Mitochondrien mit zahlreichen linearen parakristallinen Strukturen. Die Myofibrillen sind regelrecht konfiguriert. Transmissionselektronenmikroskopie, Vergr. 7100:1. **b** Detailaufnahme der Mitochondrien im Myokard mit zahlreichen parakristallinen Inklusionen. Transmissionselektronenmikroskopie, Vergr. 44.000:1. **c** Hepatozyt mit Lipidvesikeln. Aufgrund autolytischer Veränderungen ließen sich hier die Mitochondrien nicht gut darstellen. Transmissionselektronenmikroskopie, Vergr. 7100:1

Da immer noch der Verdacht auf eine Stoffwechselerkrankung bestand, wurden elektronenmikroskopische Untersuchungen des Myokards und der Leber durchgeführt (Abb. 2).

In der Leber konnte die mikrovesikuläre Steatose bestätigt werden. Anhaltspunkte für eine Stoffwechselerkrankung, insbesondere eine Glykogenose oder auch eine Sphingolipidose fanden sich nicht. Die Myokardiozyten zeigten einen regelrechten Aufbau mit erhaltenen Myofilamenten. Es zeigte sich jedoch eine Zunahme der Mitochondrienanzahl. Zudem fanden sich in fast allen Mitochondrien lineare parakristalline Einschlüsse.

In den in der Zwischenzeit vorliegenden Ergebnissen des umfangreichen Stoffwechsel- und Infektionsscreenings zeigten sich keine auffälligen Befunde.

Die morphologischen Befunde legen zusammenfassend klar eine Mitochondriopathie nahe. Die beschriebene mikrovesikuläre Steatose ist zwar per se unspezifisch, sehr häufig handelt es sich jedoch um ein indirektes Zeichen einer Schädigung/Veränderung der mitochondrialen Beta-Oxidation.

Als Todesursache des Kindes wurde schließlich unter Berücksichtigung der Klinik eine Hypoxie im Rahmen eines Kreislaufversagens postuliert. Es wurde empfohlen, die Familie auf das Vorliegen einer Mitochondriopathie abklären zu lassen.

Diese Abklärungen wurden im Rahmen einer Trio-Exom-Analyse (Untersuchung des Exoms von Vater, Mutter und Kind) durchgeführt. Hierbei ließen sich folgende Varianten nachweisen:

- TYMP c.585C>A, p.(Asp195Glu): heterozygot maternal vererbt; Einschätzung: Variante unklarer Signifikanz (VUS)
- AIFM1 (XLR) c.1646C>T, p.(Ala549Val): hemizygot, maternal vererbt; Einschätzung: Variante unklarer Signifikanz (VUS)
- GCDH (AR) c.1240G>A, p.(Glu414Lys): heterozygot, paternal vererbt; Einschätzung: pathogene Mutation

Die Untersuchung der mitochondrialen DNA (mtDNA) ist bislang aufgrund des nur eingeschränkt verfügbaren Materials zurückgestellt worden.

## Diskussion

Dieser Fall zeigt gut die multimodale Rolle der Autopsie auf, wenn es darum geht, unbekannte bzw. unerkannte Befunde zu entdecken bzw. zu erklären. Die immer mehr zurückgedrängte Technik der Autopsie erlebte in der COVID-19-Pandemie einen Wiederaufstieg, da sie half, die Pathogenese der SARS-CoV-2-Infektion besser zu verstehen, und so auch Ansätze für Therapieoptionen gab [3, 4].

Mitochondriopathien stellen ein sehr heterogenes Krankheitsbild dar, das sich klinisch in mannigfaltiger Weise präsentieren kann. Auch morphologisch können sich hier sehr diverse Befunde ergeben. Goldstandard der Diagnostik ist eine entsprechende genetische Abklärung der Patienten unter Einschluss der Klinik und der (Familien‑)Anamnese [1]. Die molekulargenetische Untersuchung beinhaltet die Analyse der nukleären und der mitochondrialen DNA. Aufgrund des Heteroplasmiegrades der mitochondrialen DNA können die Untersuchungen teilweise auch inkonklusiv bleiben.

Lichtmikroskopisch können zum Teil „Riesenmitochondrien“ nachgewiesen werden. Oft ist zur besseren Beurteilung zur Fragestellung einer Mitochondriopathie im pathologischen Einsendegut eine schon durch die Größe der Mitochondrien bedingte ultrastrukturelle Untersuchung mit der Transmissionselektronenmikroskopie notwendig [8]. Es können sowohl Veränderungen in der Anzahl („Pleoconie“) und Größe („Megaconie“) als auch der Struktur, insbesondere der inneren Mitochondrienmembran und der durch sie geformten Cristae nachgewiesen werden [2].

In den genetischen Untersuchungen konnten 3 Varianten beim Patienten nachgewiesen werden. Pathogene Varianten im *TYMP*-Gen gelten als ursächlich für eine autosomal-rezessiv erbliche, *TYMP*-bedingte mitochondriale neurogastrointestinale Enzephalopathie (MNGIE; https://omim.org/entry/603041). Pathogene Varianten im *AIFM1*-Gen gelten u. a. als ursächlich für einen X‑chromosomal gebundenen, AIFM1-bedingten kombinierten Mangel der oxidativen Phosphorylierung (COXPD6; https://omim.org/entry/300816). Pathogene Varianten im *GCDH*-Gen gelten als ursächlich für eine autosomal-rezessiv erbliche, GCDH-bedingte Glutarazidurie (https://omim.org/entry/231670).

Die gefundenen Varianten im *TYMP*- und *AIFM1*-Gen sind als Varianten unklarer Signifikanz (VUS) beschrieben und somit derzeit gemäß den ACMG-Kriterien als Mutationen mit unklarer klinischer Bedeutung anzusehen. Interessant ist jedoch, dass pathogene Varianten dieser Gene mit Mitochondriopathien assoziiert sein können.

*GCDH*-Mutationen verursachen die autosomal-rezessive Glutarazidurie. Hierbei handelt es sich um eine neurometabolische Erkrankung, die zu einer raschen Degeneration des Striatums führt. In-vitro-Studien an HeLa-Zellen mit entsprechenden *GCDH*-Mutationen konnten morphologische Veränderungen in Mitochondrien induzieren, die den bei unserem Patienten nachgewiesenen Veränderungen ähneln [6]. Dies wurde bei Substitutionen durch die Aminosäuren Alanin, Lysin und Methionin, nicht aber bei einer Substitution durch Histidin oder Leucin beobachtet. Auch wenn die Mutation unseres Patienten nicht in der Studie vertreten war, könnte die Tatsache, dass auch bei uns eine Substitution von Glutamat durch Lysin vorlag, einen Hinweis darauf geben, dass doch die *GCDH*-Mutation etwas mit der in zahlreichen Mitochondrien beobachteten Änderung der inneren Mitochondrienmembran zu tun haben könnte – natürlich mit dem Caveat, dass bei unserem Patienten ja nur eines der *GCDH*-Allele die Mutation aufwies. Neurologische Symptome waren weder beim Patienten noch bei seinem Vater bekannt. Interessanterweise konnte auch hier an homozygoten Knock-out-Mäusen gezeigt werden, dass eine Glutaryl-CoA-Dehydrogenase-Defizienz zu Veränderungen der Kommunikation zwischen Mitochondrien und dem endoplasmatischen Retikulum führt und den oxidativen Stress erhöhen kann [7].

Obwohl es somit nicht möglich ist, eine klare Diagnose zu formulieren, zeigen die erwähnten Maus- und Zellkulturversuche, dass die erwähnten Gene und strukturelle Veränderungen der Mitochondrien miteinander korrelieren. Ob hier nun das Aufeinandertreffen der Mutationen die beschriebenen mitochondrialen Strukturveränderungen und somit das Versterben des Kindes ausgelöst haben, lässt sich zum jetzigen Zeitpunkt nicht mit Sicherheit beantworten, es bleibt jedoch im Bereich des Möglichen.

## Fazit für die Praxis

- Die Autopsie hat bei der Klärung einer unklaren Todesursache auch im 21. Jahrhundert eine wichtige Stellung.
- Insbesondere bei Kindern, die unerwartet versterben, kann eine konsequente morphologische Aufarbeitung (inklusive ultrastruktureller Untersuchungen) bisher unerwartete Befunde zeigen, die entsprechende weitere Abklärungen nach sich ziehen und die auch für die Familienmitglieder eine große Relevanz haben können.
